# Highly Synchronized Expression of Lineage-Specific Genes during* In Vitro* Hepatic Differentiation of Human Pluripotent Stem Cell Lines

**DOI:** 10.1155/2016/8648356

**Published:** 2016-02-01

**Authors:** Nidal Ghosheh, Björn Olsson, Josefina Edsbagge, Barbara Küppers-Munther, Mariska Van Giezen, Annika Asplund, Tommy B. Andersson, Petter Björquist, Helena Carén, Stina Simonsson, Peter Sartipy, Jane Synnergren

**Affiliations:** ^1^School of Bioscience, Systems Biology Research Center, University of Skövde, 541 28 Skövde, Sweden; ^2^Institute of Biomedicine, Department of Clinical Chemistry and Transfusion Medicine, Sahlgrenska Academy, University of Gothenburg, 413 45 Gothenburg, Sweden; ^3^Takara Bio Europe AB, Arvid Wallgrens Backe 20, 413 46 Gothenburg, Sweden; ^4^AstraZeneca R&D, CVMD DMPK, 431 83 Mölndal, Sweden; ^5^Department of Physiology and Pharmacology, Section of Pharmacogenetics, Karolinska Institutet, 171 77 Stockholm, Sweden; ^6^NovaHep AB, Arvid Wallgrens Backe 20, 413 46 Gothenburg, Sweden; ^7^Sahlgrenska Cancer Center, Department of Pathology, Institute of Biomedicine, Sahlgrenska Academy, University of Gothenburg, 405 30 Gothenburg, Sweden; ^8^AstraZeneca R&D, GMD CVMD GMed, 431 83 Mölndal, Sweden

## Abstract

Human pluripotent stem cells- (hPSCs-) derived hepatocytes have the potential to replace many hepatic models in drug discovery and provide a cell source for regenerative medicine applications. However, the generation of fully functional hPSC-derived hepatocytes is still a challenge. Towards gaining better understanding of the differentiation and maturation process, we employed a standardized protocol to differentiate six hPSC lines into hepatocytes and investigated the synchronicity of the hPSC lines by applying RT-qPCR to assess the expression of lineage-specific genes (*OCT4, NANOG, T, SOX17, CXCR4, CER1, HHEX, TBX3, PROX1, HNF6, AFP, HNF4a, KRT18, ALB, AAT*, and* CYP3A4*) which serve as markers for different stages during liver development. The data was evaluated using correlation and clustering analysis, demonstrating that the expression of these markers is highly synchronized and correlated well across all cell lines. The analysis also revealed a distribution of the markers in groups reflecting the developmental stages of hepatocytes. Functional analysis of the differentiated cells further confirmed their hepatic phenotype. Taken together, these results demonstrate, on the molecular level, the highly synchronized differentiation pattern across multiple hPSC lines. Moreover, this study provides additional understanding for future efforts to improve the functionality of hPSC-derived hepatocytes and thereby increase the value of related models.

## 1. Introduction

The generation of a clinically relevant* in vitro* hepatocyte experimental model is challenging since the model has to mirror the diverse properties and functionality of its* in vivo* counterpart [1]. At present, primary cells isolated from human livers are considered to be the best* in vitro* hepatocyte model [2]. However, these cells still have several limitations, such as shortage in availability, rapid phenotypical changes following their isolation, and* in vitro* manipulation including decreased hepatic enzyme functionality, short life span, substantial interindividual variations, and lack of bile collection [2–4]. Thus, the pursuit of a better candidate model is strongly motivated. In this regard, human pluripotent stem cells (hPSCs), characterized by their unique capacities of self-renewal and differentiation, may provide an attractive alternative. These cells constitute an excellent human cell source for use in basic research and drug discovery and also potentially in future regenerative medicine and cell therapy applications. Moreover, the use of human induced pluripotent stem cells (hiPSCs), which are stem cells derived from reprogrammed somatic cells, enables the development of disease models and studies of interindividual diversity in safety pharmacology and toxicology [5, 6]. However, in order to fully realize the great potential of these cells, robust differentiation protocols are required to ensure reproducibility and recapitulation of the mature hepatic functionality in the final cell population [7]. Recent reports have indeed demonstrated efficient differentiation of hPSCs into hepatocytes that share many features of their* in vivo* counterparts, including the expression of hepatic markers and genes involved in drug metabolism and transport [8–11]. In addition, the cells have shown the ability to accurately predict and classify the toxicity of various compounds [6, 12].

Although the results from the hPSC-differentiation are encouraging, establishment of* in vivo*-like functionality of the* in vitro*-derived hepatocytes has still not been achieved, mainly due to impaired expression of key genes, which are critical for the metabolic functionality of the cells, a limitation that inhibits their utility in some applications in therapeutics and drug discovery [5]. Recently, Asplund and coworkers reported a standardized protocol to generate homogenous hepatocyte-like cell cultures from panel hPSC lines, which displayed metabolic diversity reminiscent of intraindividual variation found in the human population. That study showed notable similarities between the large number of cell lines analyzed but also variability of hepatic enzyme activity including CYP1A, CYP2C9, CYP2D6, and CYP3A [13]. The majority of previously reported protocols for differentiating hPSCs to hepatocyte-like cells recapitulate liver development, where the cells are first differentiated to definitive endoderm (DE) and subsequently to hepatoblast (the hepatic progenitor that gives rise to both hepatocytes and biliary cells) and finally to hepatocyte-like cells [7, 13–15]. The growth and differentiation of hepatocytes during embryonic liver organogenesis is known to be highly synchronized [16]. However, whether biological replicates from multiple hPSC lines are synchronized through an* in vitro* hepatocyte differentiation process has not been thoroughly investigated. Synchronicity accounts for the robustness of the differentiation protocol in recapitulating liver organogenesis* in vitro*, and thus the onset time of the different developmental stages for biological replicates should be consistent.

In this study, we have investigated the expression of a selected set of lineage-specific genes during the differentiation of six hPSC lines, including three hESC and three hiPSC lines, to hepatocyte-like cells applying a protocol further developed from the procedure reported by Asplund and coworkers [13]. Sixteen key genes were analyzed by reverse transcription quantitative real-time PCR (RT-qPCR):* OCT4* and* NANOG* as pluripotent markers;* T* (Brachyury) as primitive streak marker;* CXCR4, SOX17*, and* CER1* as definitive endoderm markers;* HHEX* as ventral foregut endoderm marker [17];* PROX1, TBX3*, and* HNF6* as hepatoblast markers;* AFP* as fetal hepatocyte marker; and* HNF4A* (HNF4a),* CYP3A4*,* SERPINA1* (AAT),* ALB* (albumin), and* KRT18* (CK18) as hepatic markers [14]. The RT-qPCR results were statistically analyzed using Spearman's rank correlation. A clustering analysis was also performed based on the gene expression values. The results presented here show highly synchronized and correlated gene expression profiles across the six cell lines. In addition, the functionality of mature hepatocytes-like cells was confirmed by measuring the drug metabolizing activity of Cytochrome P450 (CYP) enzymes CYP1A, CYP3A, CYP2C9, CYP2D6, and CYP2C19. Furthermore, these cells have the ability to store glycogen and they express the drug transporters MRP2, OATP1B1, NTCP, and BSEP. Interestingly, the hESC or hiPSC lines did not show any pattern indicating any specific correlation to each other. Furthermore, the clustering analysis shows the distribution of lineage-specific markers in groups, reflecting the differentiation stages of hepatocytes.

## 2. Materials and Methods

### 2.1. Human Pluripotent Stem Cell Culture and Differentiation

All hPSC lines used in this study are XY and were provided by Takara Clontech (http://www.clontech.com). The cells were thawed, maintained, and passaged in the feeder-free Cellartis DEF-CS culturing system (Takara Clontech) according to the manufacturer's recommendations. The cell lines were used in subsequent differentiation experiments at the following passages: Cellartis SA121 p.10, Cellartis SA181 p.11, Cellartis ChiPSC6b p.16, Cellartis AS034 p.10, Cellartis P11012 p.18, and Cellartis P11025 p.21. (Throughout this paper, the cell lines are referred to with their short names: SA121, SA181, ChiPSC6b, AS034, P11012, and P11025, resp.).

The hPSCs were differentiated into definitive endoderm (DE) cells by applying Cellartis DE differentiation kit (Takara Clontech) according to the manufacturer's recommendations.

**Figure 1:**
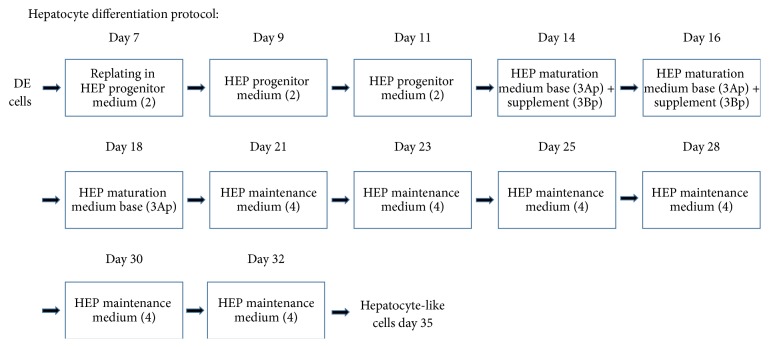
Schematic overview of the protocol used for the hepatic differentiation. The DE cells were harvested at day 7 and replated in HEP progenitor medium (2) on plates coated with the specific hepatocyte coating (1), which is included in the prototype of Cellartis Hep differentiation kit (Takara Clontech). Medium change with HEP progenitor medium (2) was performed at days 9 and 11. At days 14 and 16 the cells received HEP maturation medium base (3Ap) and supplement (3Bp). At day 18 the cells received HEP maturation medium base (3Ap). At days 21, 23, 25, 28, 30, and 32 the cells received HEP maintenance medium (4); thereafter they were maintained in culture until day 35.

At day 7, the cells were harvested according to the manufacturer's recommendations and differentiated into hepatocyte-like cells applying a prototype of the Cellartis Hep differentiation kit (available upon request from Takara Clontech) as illustrated in Figure 1.

### 2.2. RNA Extraction and Real-Time Quantitative Polymerase Chain Reaction

Cell samples were collected daily before performing medium change (if medium change was scheduled) during the differentiation process and preserved in RNAprotect Cell Reagent (Cat. number 76526, QIAGEN) at −20°C. RNA was extracted using MagMAX-96 Total RNA Isolation Kit (Cat. number AM1830, Life Technologies) and quantified by using GeneQuantpro spectrophotometer. Eighty ng RNA of each sample was used to synthesize cDNA applying the iScript cDNA Synthesis Kit (Cat. number 170-8890, BIO-RAD).

TaqMan Fast Advanced Master Mix (Cat. number 4444557, Life Technologies) and TaqMan Gene Expression Assays were used in RT-qPCR.  Table 1 summarizes the assays that were used and on which days they were applied. Each reaction included 1.6 ng cDNA and was run in duplicate.* CREBBP* was used as reference gene and an in-house calibrator was used for sample normalization as well [13]. For hepatoblast, fetal, and mature hepatocytes markers an RNA pool of freshly isolated human primary hepatocytes from 5 different donors was used as a calibrator. Interplate controls were added for plate normalization.

The difference in quantification cycle (ΔΔ*C*_*q*_) and relative quantification (RQ) were calculated according to the following formulas:

(1) ΔCqsample=Cqtarget−Cqreference,ΔΔCq=ΔCqsample−ΔCqcalibrator,RQ=2−ΔΔCq.

### 2.3. Statistical Analysis of Gene Expression Data

For each gene, the expression data consisted of a matrix *E*_*ij*_ of RQ values, where *i* = 1,…, 6 represents the six cell lines and *j* = *d*_min_,…, *d*_max_ represents the days at which expression was measured for the particular gene. For each gene, the RQ vectors of every pair of cell lines were tested for association using Spearman rank correlation, which is a nonparametric test, not sensitive to extreme values, which can be used to detect nonlinear relationships [18]. Correlation coefficients were interpreted according to a scale where 0.8–1.0 was defined as “very strong,” 0.6–0.8 as “strong,” 0.4–0.6 as “moderate,” 0.2–0.4 as “weak,” and 0.0–0.2 as “very weak” or “no correlation.”

For the clustering analysis, the matrix *E*_*ij*_ was replaced with the vector median (*E*_*j*_), where each value is the median RQ for a particular day over all six cell lines. Groups of these RQ vectors, representing different combinations of genes, were hierarchically clustered with the HCL algorithm in the MultiExperiment Viewer software v4.9 (http://www.tm4.org/mev.html), using Spearman rank correlation as similarity metric and complete linkage as the linkage rule.

### 2.4. Immunocytochemistry

At days 0, 5, 7, 14, 24, 25, 29, and 30 of differentiation, the cells were washed with DPBS (+/+) (Cat. number 14040-, Life Technologies) and fixed by incubation for 10 min in 4% formaldehyde (Cat. number 02176, Histolab, Västra Frölunda, Sweden) and then washed and maintained in DPBS (+/+) until processing. TNB-blocking buffer was prepared by mixing 0.1 M Tris (Cat. number 10421-1, Kebo Lab AB) adjusted with hydrochloric acid 36.5%–38% (Cat. number H1758, Sigma) to pH 7.5 with 0.15 M NaCl (Cat. number S5886, Sigma) in dH_2_O. Blocking reagent from Perkin Elmer TSA-kit (Cat. number FP1012, Perkin Elmer) was slowly added to Tris-HCl/NaCl buffer under stirring to final concentration of 0.5%; then the solution was heated to 60°C until the blocking reagent was dissolved. The cells were washed once with DPBS (+/+), followed by incubation for 10 min in 0.3% Triton-X (Cat. number T8532, Sigma) in DPBS (+/+), and the cells were subsequently incubated in TNB-blocking buffer for 1 h. Primary antibodies diluted in 0.1% Triton-X in DPBS (+/+) were added to the cultures and incubated over night at 4°C. The following day, the cells were washed three times with DPBS (+/+); then the secondary antibodies and DAPI diluted in 0.1% Triton-X in DPBS (+/+) were added and the cells were incubated for 2 h at RT. Finally, the cells were washed three times with DPBS (+/+) before being imaged in a fluorescence microscope and photographed. The photos were processed using ImageJ software (http://imagej.nih.gov/ij/).  Table 2 summarizes the different primary and secondary antibodies applied, the dilution ratios, and the days at which respective markers were analyzed.

### 2.5. Cytochrome P450 (CYP) Enzymes Activity Assay

The activities of the enzymes CYP1A, CYP3A, CYP2C9, CYP2C19, and CYP2D6 in hepatocyte-like cells differentiated from all hPSC lines used in this study and in cryoplateable human primary hepatocytes from four different donors (BioreclamationIVT, Frankfurt am Main, Germany) were measured as described previously with small modification [13]. Briefly, hPSC-derived hepatocyte-like cells at day 29 of the hepatic differentiation and primary human hepatocytes (cultured for in total 20 h after plating) were incubated in a cocktail of CYP substrates, 10 *μ*M phenacetin a CYP1A substrate, 10 *μ*M bufuralol a CYP2D6 substrate, 10 *μ*M diclofenac a substrate of CYP2C9, 50 *μ*M mephenytoin a substrate of CYP2C19, and 5 *μ*M midazolam a substrate of CYP3A. The formation of the metabolites (paracetamol (CYP1A), 1-OH-bufuralol (CYP2D6), 4-OH-diclofenac (CYP2C9), 4-OH-mephenytoin (CYP2C19), and 3-OH-midazolam (CYP3A)) was determined by liquid chromatography/mass spectrometry (LC/MS) performed at Pharmacelsus GmbH (Saarbrücken, Germany). The samples were collected in duplicate for each cell line, and the metabolite concentration was normalized to the protein amount per well and incubation time and the results are presented as pmol metabolite/mg protein/min.

### 2.6. PAS Staining

The cells were fixed as described above. A PAS kit was applied (395B-1KT, Sigma Aldrich), in which the cells were incubated in periodic acid for 15 min on a shaker at RT. Then the cells were washed with dH_2_O and incubated in SCHIFF reagent for 30 min on a shaker at RT. Subsequently the cells were washed with dH_2_O and incubated in hematoxylin for 90 seconds and finally the cells were washed with dH_2_O again.

## 3. Results

### 3.1. Human PSC Culturing and Hepatic Differentiation

**Figure 2:**
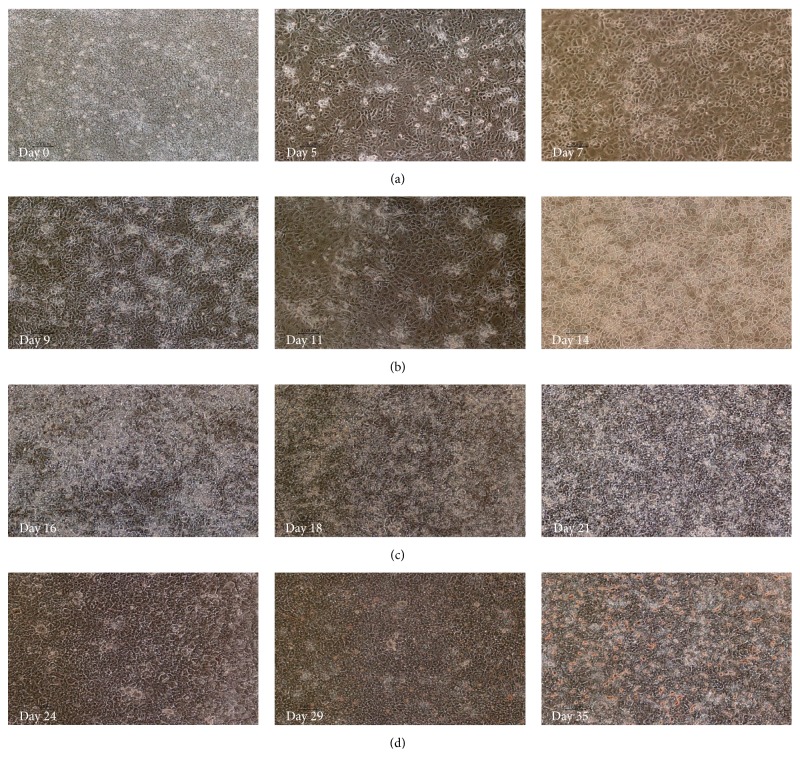
Morphology of cells during hepatic differentiation at days 0, 5, 7, 9 (ChiPSC6b), 11 (P11012), 14 (ChiPSC6b), 16 (P11012), 18 (P11012), 21 (P11012), 24 (SA121), 29 (AS034), and 35 (ChiPSC6b) magnification 10x. (a) Human PSCs cultured in Cellartis DEF-CS demonstrate stem cell morphology at day 0 (AS034). (b) At day 5 (P11025) and day 7 (SA181) the cells show a characteristic DE morphology. (c) Following the application of HEP progenitor medium, the DE cells transferred gradually to hepatoblasts. (d) The cells acquired the hepatocyte morphology (polygonal cells) following the application of HEP maturation medium (3Ap) and supplement (3Bp). In the HEP maintenance medium (4) the cells preserve typical hepatocyte morphology, polygonal cells with single- or binucleus.

Human PSCs cultured in the Cellartis DEF-CS culturing system showed typical stem cell morphology (round cells with big nucleus) prior to harvesting at day 0 (Figure 2(a)). The cell morphology started to change with the application of Cellartis DE differentiation kit. At day 5 the cells showed some morphology indicative of DE, and on day 7 the shape of the cells became more spikey or triangular, which is the typical DE morphology having reached confluence (Figure 2(a)). The cells were dissociated and replated, and hepatocyte differentiation was performed by applying a prototype of Cellartis Hep differentiation kit. The hepatocyte differentiation protocol is schematically described in Figure 1. The typical DE cell morphology changed during the application of HEP progenitor medium (Figure 2(b)) and after the application of HEP maturation medium the cells gradually acquired the hepatocyte morphology (Figure 2(c)). Finally, when the cells were cultured in HEP maintenance medium, they acquired the typical hepatocyte morphology, and polygonal single- or binucleated cells were observed (Figure 2(d)).

### 3.2. Gene Expression of Lineage-Specific Markers

**Figure 3:**
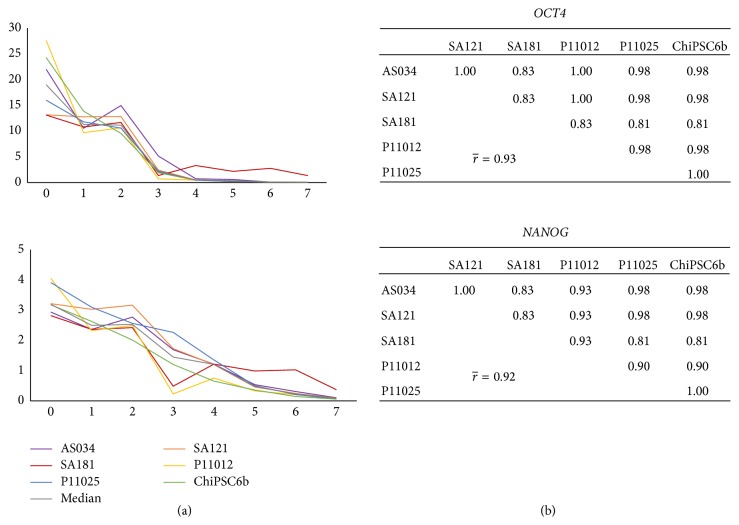
Gene expression profiles of the pluripotent markers* OCT4* and* NANOG*. (a) The *x*-axis indicates the days during differentiation and the *y*-axis indicates relative quantification (RQ) where the calibrator's RQ = 1. The colored lines show the results from each individual hPSC line and the grey line indicates the calculated median value of the RNA level. (b) Correlation tables show very strong correlations between the cell lines, with an average pairwise correlation of 0.93 for* OCT4* and 0.92 for* NANOG* for the interval of day 0 to day 7.

**Figure 4:**
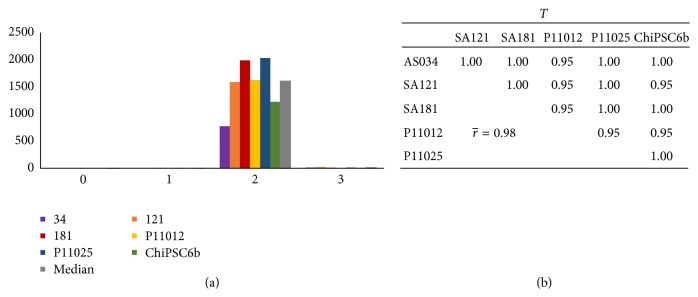
(a) Bar graph showing the RNA expression levels of the primitive streak marker *T*. The *x*-axis indicates the day of differentiation and the *y*-axis indicates RQ, where the calibrator's RQ = 1. All six cell lines express the *T* gene essentially only at day 2; therefore, the correlation between cell lines for the interval of day 0 to day 3 is very strong as indicated in (b).

**Figure 5:**
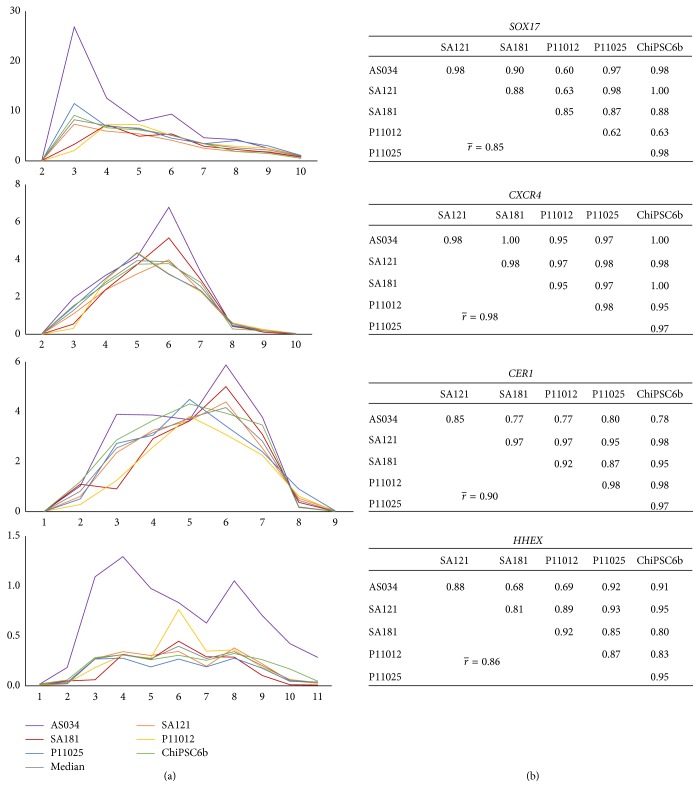
Gene expression profiles for DE (*SOX17*,* CXCR4*, and* CER1*) and ventral foregut endoderm (*HHEX*) markers in the interval from day 1 to day 11. (a) The *x*-axis indicates the days during differentiation and the *y*-axis indicates RQ, where the calibrator's RQ = 1. The colored lines show the results from each individual hPSC line and the grey line indicates the calculated median value of the RNA level. Correlation tables are shown in (b). The time points before the onset of expression and after the RNA levels have decreased below the detection limit are not included in the analysis (e.g., day 1 and day 11 were excluded for* SOX17*). In general, the correlation between cell lines for all these markers is very strong. DE cells seem to emerge at day 3 and disappear at day 8.

For each hPSC line, RNA was collected daily throughout the differentiation process and subsequently analyzed using RT-qPCR for the lineage-specific markers listed in Table 2.  Figure 3 shows the relative quantification curves for the early stages markers, in addition to pairwise cell line correlation tables for each marker. The pluripotency markers* OCT4* and* NANOG* were distinctly downregulated early during the differentiation process;* OCT4* was expressed below the detection limit (*C*_*q*_ ≥ 35) already at day 4 and* NANOG* at day 7. The average pairwise correlation coefficients between cell lines for these two markers are > 0.9 (Figure 3), indicating very high synchroneity among the cell lines at this stage. The expression of* T* (Brachyury), a pan-mesoderm marker expressed in mesendoderm which is a precursor of both mesoderm and endoderm [19], peaks at day 2 in all cell lines (Figure 4).  Figure 5 shows the onset of the DE markers* SOX17* and* CXCR4* at day 3 and the average correlation coefficients for these markers during day 2 to day 10 are very high (0.85 and 0.98, resp.). The expression of* SOX17* decreased gradually until it fell below the detection limit at day 10. The expression of* CXCR4* was upregulated until day 5 or day 6, and then it was downregulated and subsequently undetectable at day 9. The onset of the DE marker* CER1* occurred already at the mesendoderm stage (day 2), one day earlier than* SOX17* and* CXCR4*, and its expression increased until day 6 after which it started to decrease and, similar to* CXCR4*, it was below the detection limit at day 9. The average correlation coefficient for* CER1* is very strong (0.90) and there is no substantial difference in* CER1* expression between the cell lines.* HHEX*, a marker for ventral foregut endoderm, was also expressed at the DE stage and subsequently undetectable at day 11 (except for line AS034), with a very strong average correlation coefficient of 0.86 as shown in Figure 5. In general, the correlation analysis does not show any preference of hESC lines to correlate with each other rather than with hiPSC lines or vice versa (Figures 3, 4, and 5).

**Figure 6:**
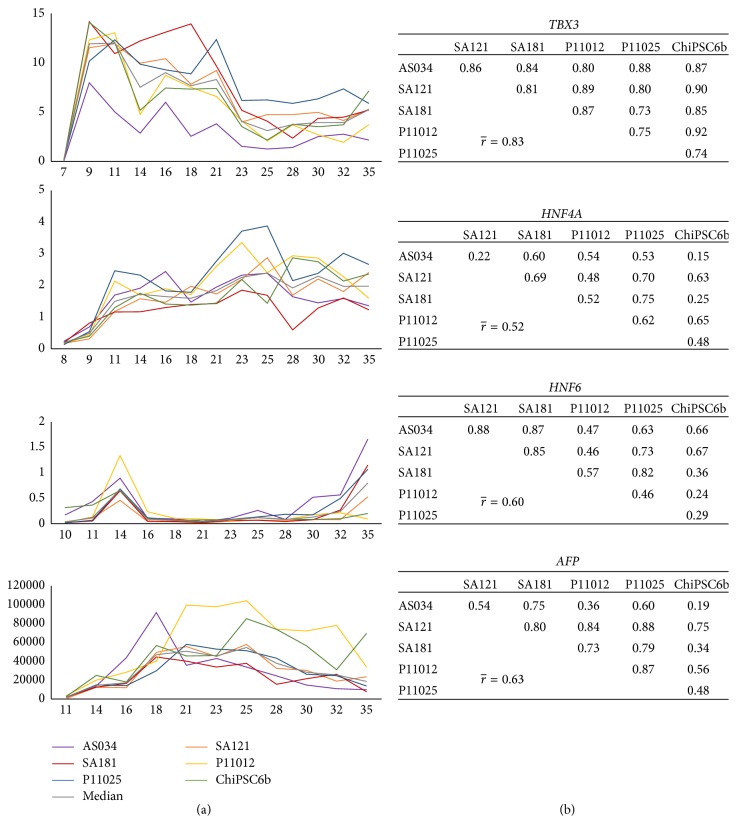
Gene expression profiles of early hepatic markers (*TBX3*,* HNF4A*,* HNF6*, and* AFP*) in the interval from day 7 to day 35. (a) The *x*-axis indicates the days during differentiation and the *y*-axis indicates RQ, where the calibrator's RQ = 1. The colored lines show the results from each individual hPSC line and the grey line indicates the calculated median value of the RNA level. Correlation tables are shown in (b). The graphs show synchronicity of onset as well as up- and downregulation among all cell lines for the genes* TBX3*,* HNF4A*,* HNF6*, and AFP. The average correlation between pairs of cell lines ranges from moderate (0.52 for* HNF4A*) to very strong (0.83 for* TBX3*).

**Figure 7:**
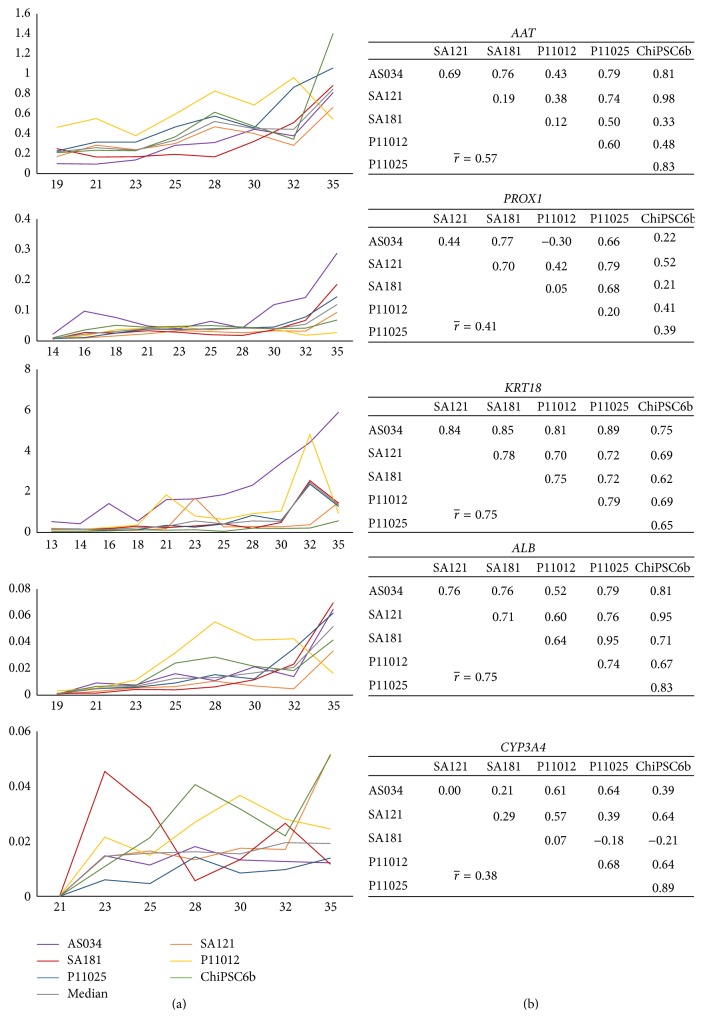
Gene expression profiles of later hepatic markers (*AAT*,* PROX1*,* KRT18*,* ALB*, and* CYP3A4*) in the interval from day 13 to day 35. (a) The *x*-axis indicates the days during differentiation and the *y*-axis indicates RQ, where the calibrator's RQ = 1. The colored lines show the results from each individual hPSC line and the grey line indicates the calculated median value of the RNA level. Correlation tables are shown in (b). The graphs show synchronicity of onset and up- and downregulation among all cell lines for the genes* AAT*,* PROX1*,* KRT18*,* ALB*, and* CYP3A4*. The average correlation between pairs of cell lines ranges from weak (0.38 for* CYP3A4*) to strong (0.75 for* KRT18* and* ALB*).

The correlation between cell lines regarding markers of stages following the DE phase of the hepatic differentiation is slightly lower compared to the early markers (Figures 6 and 7). Due to transient fluctuations in the RNA levels on the days after each medium change, days 8, 10, 12, 13, 15, 17, 19, 20, 22, 24, 26, 27, 29, 31, 33, and 34 were not included in the correlation analysis. The genes* TBX3*,* HNF4a*,* HNF6*, and* AFP* were expressed already at the hepatoblast stage. These genes demonstrated gene regulation synchronicity among all cell lines and, as shown in Figure 6, the average pairwise correlation ranges from moderate (0.52 for HNF4a) through strong (0.60 for* HNF6* and 0.63 for* AFP*) to very strong (0.83 for* TBX3*). The correlation of the mature hepatocyte markers ranges from weak (0.38 for* CYP3A4*), through moderate (0.41 for* PROX1* and 0.57 for* AAT*), to strong (0.75 for both* KRT18* and* ALB*) (Figure 7). Although the weakest average correlation was shown for* CYP3A4*, there are some strong and very strong correlations between pairs of cell lines for this gene, for example, 0.89 between ChiPSC6b and P11025 (Figure 7). Interestingly, and as noted above for the DE stage, there is no preference for hESC lines to correlate with each other rather than with hiPSC lines at later stages either, and the same applies to the hiPSC lines. The expression profiles for the different genes show minor fluctuations during the hepatic differentiation; however, the distinct changes in gene expression occurred in general at the same time for the different cell line, further underscoring the synchronized differentiation process across the cell lines.

### 3.3. Immunocytochemical Analysis of Lineage-Specific Markers during Hepatic Differentiation

**Figure 8:**
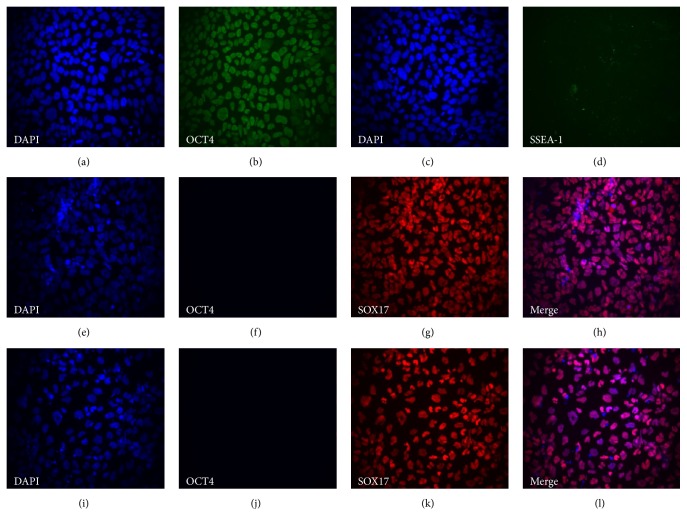
Representative micrographs from immunocytochemical analysis at days 0, 5, and 7 during hepatic differentiation of the cell line AS034. Magnification: 20x. Panels (a)–(d) show staining of undifferentiated hPSC at day 0: (a) and (c) show nuclear DAPI staining, (b) shows Oct4 staining, and (d) shows SSEA-1 staining. Panels (e)–(h) show staining of DE cells at day 5 of differentiation: (e) shows nuclear DAPI staining, (f) shows Oct4 staining, (g) shows Sox17 staining, and (h) shows a merge of DAPI and Sox17 staining. Panels (i)–(l) show staining of DE cells at day 7 of differentiation: (i) shows nuclear DAPI staining, (j) shows Oct4 staining, (k) shows Sox17 staining, and (l) shows a merge of DAPI and Sox17 staining.

The protein expression of selected markers from the different developmental stages was analyzed by immunocytochemistry.  Figure 8 shows the homogenous expression of the pluripotent marker Oct4 in hPSCs in addition to the absence of the differentiation marker SSEA-1 (Figures 8(a)–8(d)), demonstrating the undifferentiated state of the hPSC at the start of the differentiation protocol. At days 5 and 7, all cells expressed the DE marker Sox17 and the pluripotency marker Oct4 was absent (Figures 8(e)–8(h) and 8(i)–8(l), resp.), illustrating the high efficiency in generating DE cells using the differentiation kit.

**Figure 9:**
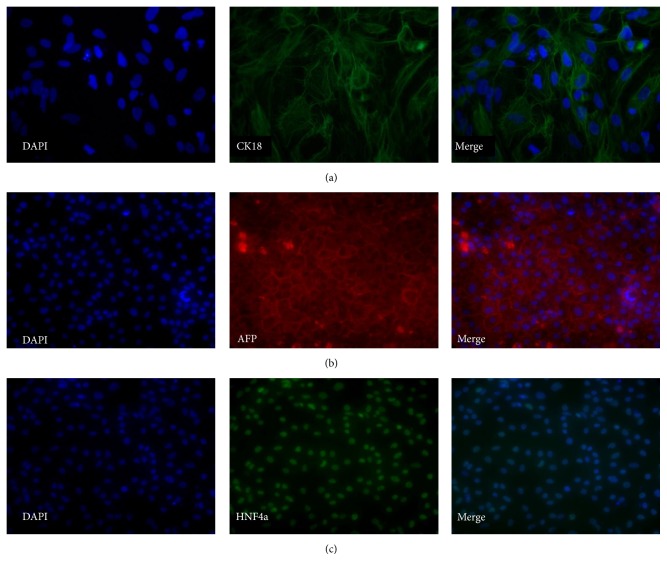
Representative micrographs illustrating the expression of selected markers at day 14 of the hepatic differentiation. Panel (a) shows staining for CK18 (SA121, 40x magnification). Panel (b) shows staining for AFP (AS034, 20x magnification). Panel (c) shows staining for HNF4a (AS034, 20x magnification).

During the differentiation from DE cells to hepatocytes, hepatic markers were gradually upregulated.  Figure 9 shows the expression of CK18, AFP, and HNF4a at day 14. The hepatic marker CK18 was weakly expressed (Figure 9(a)), while AFP and HNF4a were strongly expressed in most of the cells (Figures 9(b) and 9(c), resp.).

**Figure 10:**
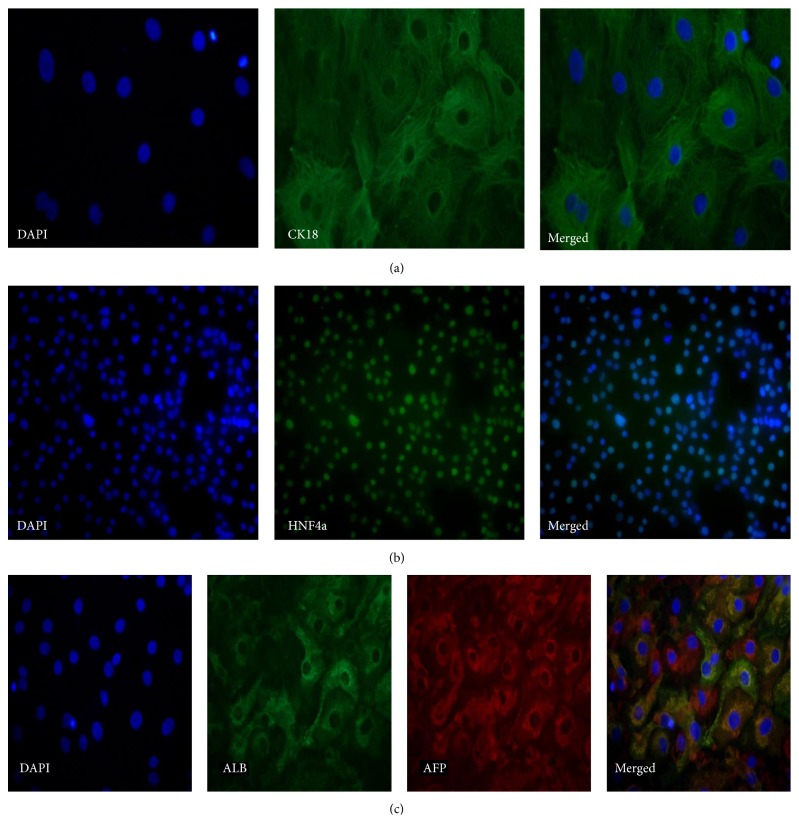
Representative micrographs illustrating the expression of markers at day 25. Panel (a) shows staining for CK18 (ChiPSC6b, 40x magnification). Panel (b) shows staining for HNF4a (AS034, 20x magnification). Panel (c) shows stainings for ALB and AFP (ChiPSC6b, 20x magnification).

At day 25, the differentiation has progressed and more mature hepatocyte markers were expressed in the cells.  Figure 10(a) shows the expression of CK18, which was remarkably denser at day 25 than at day 14. HNF4a was still expressed at day 24 at a similar level as on day 14 (Figure 10(b)). Coexpression of ALB and AFP at day 25 was detected in some hepatocyte-like cells; however, the cell cultures were heterogeneous since in some cells only AFP was expressed, which indicated a fetal-like phenotype (Figure 10(c)).

**Figure 11:**
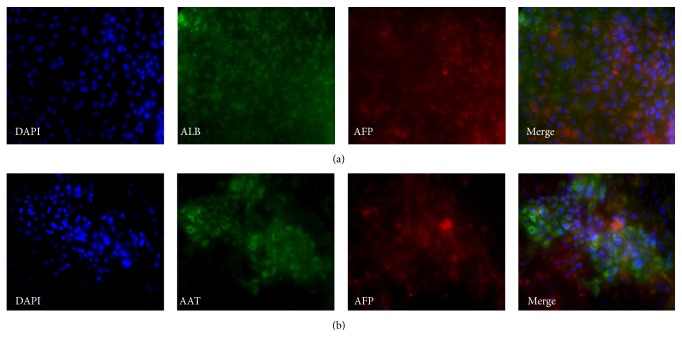
Representative micrographs illustrating the expression of markers at day 29 of the hepatic differentiation of the cell line ChiPSC6b. Magnification: 20x. Panel (a) shows stainings for ALB and AFP. Panel (b) shows stainings for AAT and AFP.

**Figure 12:**
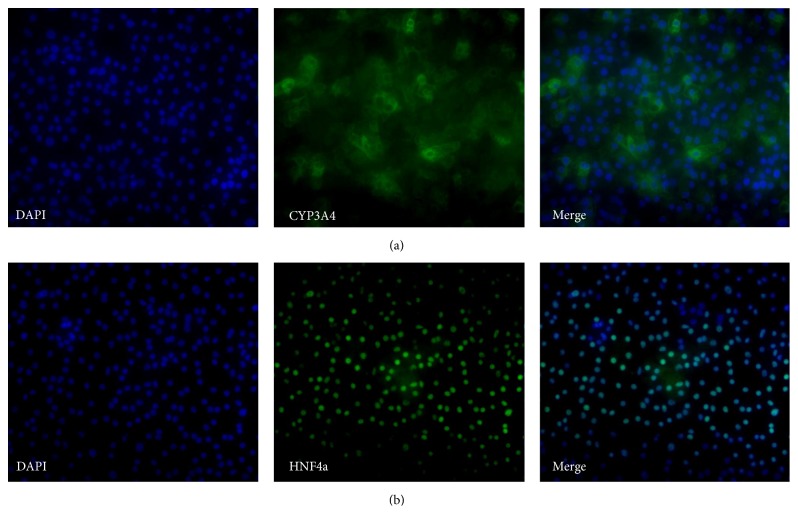
Representative micrographs illustrating the expression of markers at day 30. Panel (a) shows stainings for CYP3A4 (AS034, 20x magnification). Panel (b) shows stainings for HNF4a (AS034, 20x magnification).

At day 29, expression of AFP was still present although it was weaker than at day 25. At this stage, the cells were still heterogeneous with about 50% of the cells expressing only ALB and the remaining 50% coexpressing AFP and ALB (Figure 11(a)) indicating the immaturity of some cells at this point. Coexpression of AAT and AFP was also detected (Figure 11(b)); however, there were still some cells that only expressed AFP. At day 30, mature hepatic markers were expressed, such as CYP3A4 (Figure 12(a)); however, not all cells were immunopositive. In addition, HNF4a was still expressed in all hepatocyte-like cells (Figure 12(b)).

**Figure 13:**
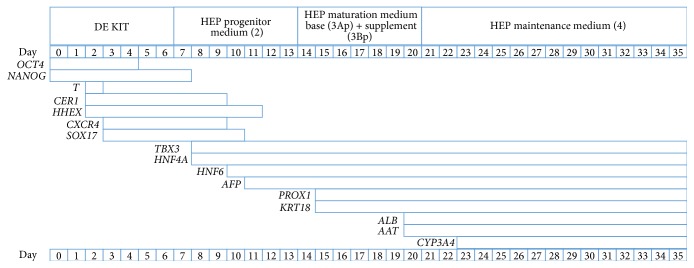
Overview of the expression patterns of all genes analyzed in this study. The header bar shows the medium in which the cells were incubated during the indicated days through hepatic differentiation. The bars below indicate the expression duration of the different genes during the hepatic differentiation. Gene expression after day 35 was not analyzed; hence end of expression for genes expressed at day 35 should not be assumed.

Figure 13 shows an overview of the expression patterns of all the genes analyzed in this study, considering genes as being expressed if *C*_*q*_ < 35. The culturing medium of the different stages during hepatic differentiation as well as the days after start of differentiation is indicated in the upper bars. The pluripotency genes,* OCT4* and* NANOG*, were expressed from day 0 and their expression partly overlapped the DE stage. The *T* gene (Brachyury) was only expressed at day 2 indicating the mesendoderm stage. The genes* CER1* and* HHEX* were initiated already at day 2 and* CXCR4* and* SOX17* appeared at day 3, indicating the onset of the DE differentiation. Progenitor and hepatocyte markers were expressed gradually after adding HEP progenitor medium.* TBX3* and* HNF4A* were already present at day 8, which is one day after the shift to HEP progenitor medium (2).* HNF6* appeared on day 10 and the fetal hepatocyte marker* AFP* on day 11. The expression of* PROX1* and* KRT18* was detected at day 15, one day after the shift to HEP maturation medium base (3Ap) with supplement (3Bp). The hepatocyte maturation marker* AAT* was already expressed at day 18.* ALB* was present from day 20 and, notably, only a few days after the addition of HEP maintenance medium (4) the adult enzyme* CYP3A4* was expressed at day 23.

### 3.4. Clustering Analysis

**Figure 14:**
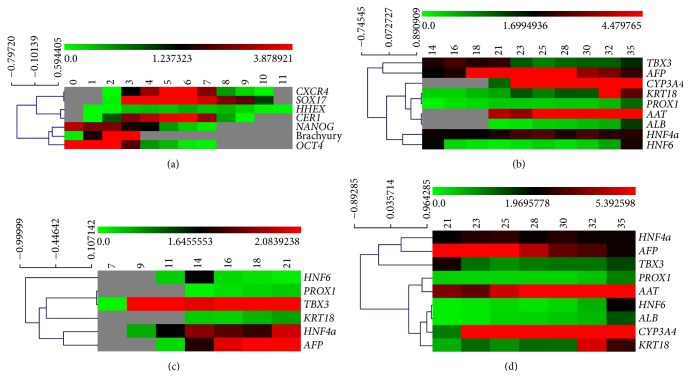
Clustering analysis. The genes included in the study were clustered applying Spearman rank correlation. Colors indicate RQ according to the scale shown above each heatmap. Missing values are indicated by gray color. (a) Clustering results for days 0 to 11, (b) days 14 to 35, (c) days 7 to 21, and (d) days 21 to 35.

In order to detect possible coregulation and similarities between all the genes that were analyzed in this study except transporters* SLC10A1* (NTCP),* ABCB11* (BSEP),* SLCO1B1* (OATP1B1), and* ABCC2* (MRP2), clustering analyses of the gene expression were performed both globally and locally for different intervals during the hepatic differentiation. The clustering of genes was performed using each gene's median expression value over all cell lines, with Spearman's rank correlation as the similarity function. The clustering of days 0 to 11 shows a clear separation between two clusters, with one containing the pluripotency markers,* OCT4* and* NANOG*, and the primitive streak marker, *T*, and the other cluster containing the DE markers* SOX17*,* CXCR4*,* CER1*,  and* HHEX*, the ventral foregut marker (Figure 14(a)). In the clustering of genes days 14 to 35 of the hepatic differentiation, the main separation is between a cluster containing the two genes* TBX3* and* AFP* and a second cluster containing the remaining genes. In the later cluster,* HNF4A* and* HNF6* are clearly separated from a subcluster containing* PROX1*,* CYP3A4*,* ALB*,* AAT*, and* KRT18* (Figure 14(b)). Notably, in the analysis of days 7 to 21,* HNF6* is clearly separated from all other genes. For the remaining genes, there were one subcluster consisting of* HNF4A* and* AFP* and a second subcluster consisting of* TBX3*,* PROX1*, and* KRT18* (Figure 14(c)). For the maturation phase (days 21 to 35) two main clusters were identified, one containing* HNF4A*,* TBX3*, and* AFP* and the other containing* PROX1*,* AAT*,* HNF6*,* KRT18*, ALB, and CYP3A4 (Figure 14(d)).

### 3.5. Gene Expression and Immunocytochemistry Analysis of Drug Transporters

**Figure 15:**
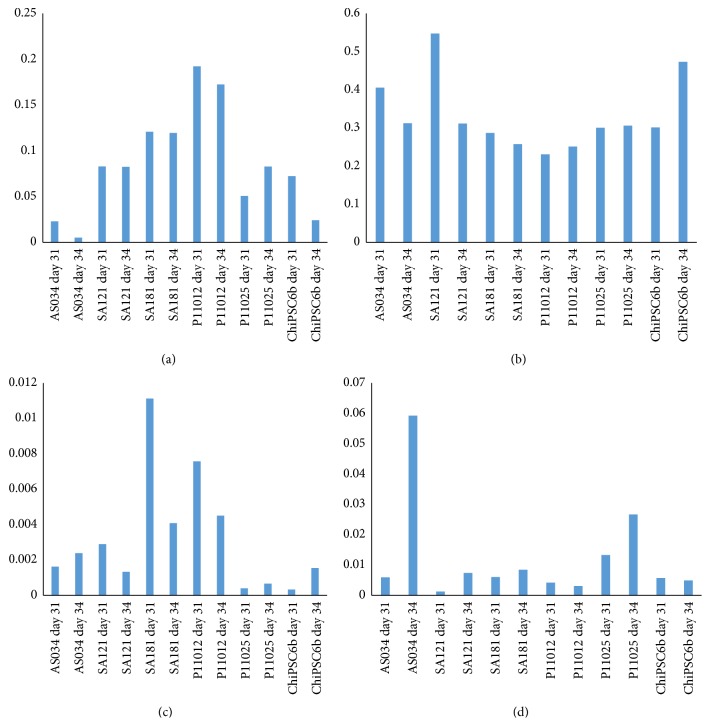
Bar graph showing the RNA expression levels of the drug transporters (a)* SLC10A1* (NTCP), (b)* ABCC2* (MRP2), (c)* SLCO1B1* (OATP1B1), and (d)* ABCB11* (BSEP). The *x*-axis indicates the hPSC lines at days 31 and 34 of the hepatic differentiation. The *y*-axis indicates RQ, where the calibrator's RQ = 1. The calibrator was an RNA pool of freshly isolated human primary hepatocytes from 5 different donors.

To determine the maturity of the hepatocyte-like cells generated from the different hPSC used in this study, RT-qPCR was applied to analyze the expression of the drug transporters* SLC10A1* (NTCP),* ABCB11* (BSEP),* SLCO1B1* (OATP1B1), and* ABCC2* (MRP2).  Figure 15 shows interindividual variation in drug transporters gene expression in hepatocyte-like cells derived from the different hPSC lines (Figure 15(a)–15(d)). In addition, the expression of NTCP (Figure 15(a)) and MRP2 (Figure 15(b)) was closer to the level of human freshly isolated primary hepatocytes (calibrator) than OATP1B1 (Figure 15(c)) and BSEP (Figure 15(d)). Most of the interindividual variation was observed for BSEP (Figure 15(d)), where hepatocyte-like cells derived from AS034 at day 34 expressed BSEP at higher levels than any of the other hPSC-derived hepatocytes. OATP1B1 also showed high interindividual variation, where hepatocytes derived from SA181 and P11012 at day 3 expressed OATP1B1 at higher levels than what were observed in the other cell lines.

**Figure 16:**
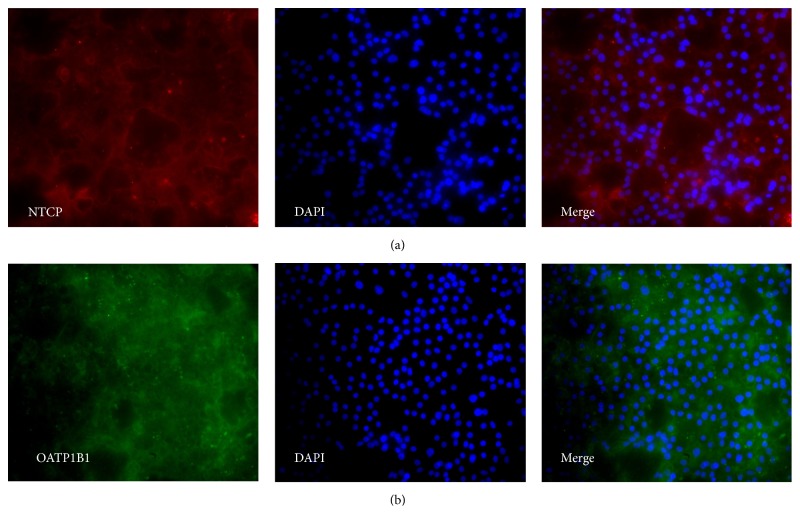
Representative micrographs illustrating the expression of drug transporters at day 29 of the hepatic differentiation of P11012. Magnification: 20x. Panel (a) shows stainings for NTCP. Panel (b) shows stainings for OATP1B1.

**Figure 17:**
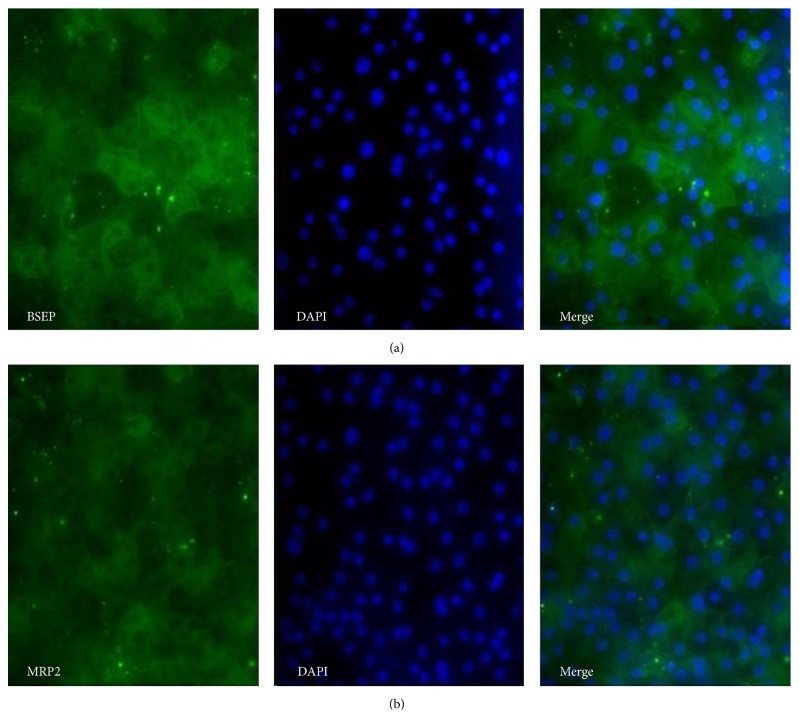
Representative micrographs illustrating the expression of drug transporters at day 29 of the hepatic differentiation. Magnification: 20x. Panel (a) shows stainings for BSEP in P11025 cell line. Panel (b) shows stainings for MRP2 in SA121 cell line.

Immunocytochemistry analyses were performed to confirm the expression of NTCP, BSEP, MRP2, and OATP1B1 at the protein level.  Figure 16 shows the staining patterns for NTCP and OATP1B1 and Figure 17 shows the expression of BSEP and MRP2.

### 3.6. Cytochrome P450 (CYP) Enzymes Activity Assay

**Figure 18:**
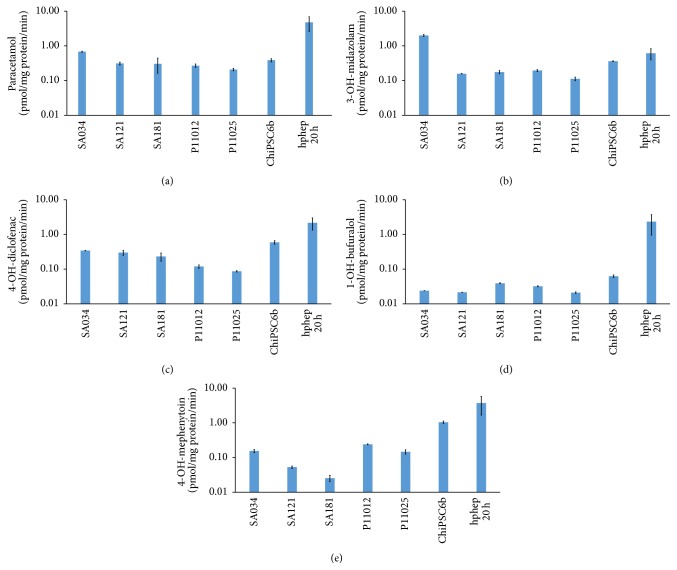
Cytochrome 450 enzymes (CYP) activities in hepatocytes derived from hPSCs (AS034, SA121, SA181, P11012, P11025, and ChiPSC6b) (*N* = 2) and cryopreserved human primary hepatocytes plated for 20 hr (hphep 20 h) (*N* = 4). CYP activities in hepatocyte cultures were measured after 29 days of differentiation by the following assay: the cells were incubated with CYP enzymes substrates phenacetin (CYP1A), midazolam (CYP3A), diclofenac (CYP2C9), bufuralol (CYP2D6), and mephenytoin (CYP2C19). The concentrations of the resulting metabolites paracetamol, 3-OH-midazolam, 4-OH-diclofenac, 1-OH-bufuralol, and 4-OH-mephenytoin were determined by liquid chromatography/mass spectrometry. The results were normalized to mg protein per well and the duration of CYP activity assay. The CYP activity is presented as pmol metabolite per mg protein per minute (mean ± SEM). The *y*-axis in log scale. (a) CYP1A, (b) CYP3A, (c) CYP2C9, (d) CYP2D6, and (e) CYP2C19.

To investigate the drug metabolizing capacity, hepatocyte-like cells and cryoplateable human primary hepatocytes (hphep) were incubated in a cocktail of phenacetin (metabolized by CYP1A), bufuralol (metabolized by CYP2D6), diclofenac (metabolized by CYP2C9), midazolam (metabolized by CYP3A), and mephenytoin (metabolized by CYP2C19) at day 29 of the hepatic differentiation. The concentrations of the resulting metabolites paracetamol, 1-OH-bufuralol, 4-OH-diclofenac, 3-OH-midazolam, and 4-OH-mephenytoin were determined by liquid chromatography/mass spectrometry. The metabolite concentrations were normalized to mg protein and incubation time.  Figure 18 shows interindividual variation in the different CYP enzyme activities in the hepatocyte-like cells generated from the different hPSC lines. The activity of CYP1A in hphep is about 7 times higher than in AS034-derived hepatocytes (Figure 18(a)). However, the activity of CYP3A in SA034-derived hepatocytes was higher than in hphep (Figure 18(b)). The activity of CYP2C9 in hphep is about 4 times higher than in ChiPSC6b-derived hepatocytes (Figure 18(c)). The activity of CYP2D6 in hphep is much higher than in hPSC-derived hepatocytes (Figure 18(d)). Moreover, the activity of CYP2C19 in hphep is about 4 times higher than ChiPSC6b-derived hepatocytes (Figure 18(e)). In addition, hepatocyte-like cells from SA034 show much high CYP1A and CYP3A activity compared to other hPSC-derived hepatocytes (Figures 18(a) and 18(b), resp.). Hepatocyte-like cells derived from ChiPSC6b show higher CYP2C9 activity compared to hepatocytes-like cells derived from the other cell lines (Figure 18(c)). These results illustrate the interindividual variation in CYP activity of hPSC-derived hepatocytes.

### 3.7. PAS Staining

**Figure 19:**
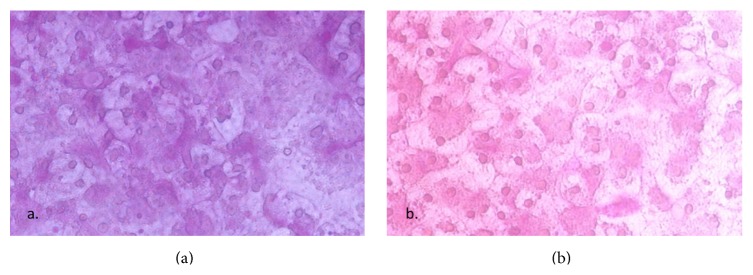
Representative PAS stainings. The cells were treated with periodic acid, SCHIFF, and hematoxylin as described in Materials and Methods. (a) P11025 and (b) AS034.

The ability to produce and store glycogen is a function of mature hepatocytes [11]. To investigate if hPSC-derived hepatocytes also have the ability to store glycogen PAS staining was performed.  Figure 19 shows the detection of glycogen storage by PAS. Hepatocytes derived from both hESC (Figure 19(b)) and hiPSC (Figure 19(a)) show glycogen storage ability.

## 4. Discussion

The unique properties of pluripotent stem cells, including their indefinite self-renewal and their capacity to differentiate to essentially all cell types in the body, make them an attractive source for human cells that can be applied in various cell models for drug discovery and future regenerative medicine applications [2, 4, 5, 7, 15, 20, 21].* In vitro* differentiation of hepatocytes, recapitulating some of the properties and functionalities of their* in vivo* counterpart, has been reported by several groups using differentiation protocols that mimic the hepatocyte development* in vivo* [4, 6–9, 15, 22]. A recent study reported a standardized hepatocyte differentiation protocol that does not require any further adjustments to produce near-homogenous hepatocytes from a large panel of different hPSC lines [13]. In the present study, we have differentiated six hPSC lines into hepatocyte-like cells applying a protocol developed further from the procedure published by Asplund and coworkers [13]. Using RT-qPCR, we analyzed the gene expression of several key lineage-specific genes that are markers for the different stages during hepatocyte differentiation to assess the synchronicity of the* in vitro* differentiation process. In addition, we applied statistical methods to mathematically analyze and quantify the correlation between the hPSC lines. The results revealed highly synchronized gene expression profiles, especially for key markers of the early differentiation stages, thus indicating the similarity of this* in vitro* differentiation process to the* in vivo* liver development. However, weak correlation of the mature hepatocyte marker* CYP3A4* could be explained by its high level of polymorphism [23]. We also noted that the correlation patterns among hESC and hiPSC lines appeared randomly distributed and no evidence of preferential similarity between specific cell lines was observed.

The hepatic differentiation process starts with the downregulation of the stem cell markers* OCT4* and* NANOG* that are also clustered together indicating similar functionality, such as maintenance of pluripotency, and both genes have been indeed reported to control the expression of each other [24].* NANOG* clustered also with* T* (Brachyury), and* NANOG* has been reported to bind the promoter region of* T* [25]. Our results also show that* OCT4* is undetectable in DE cells while* NANOG* is downregulated but still present at low levels at the DE stage, which is also consistent with the results from other studies [26, 27]. The primitive streak stage appears at day 2 with a short peak expression of* T*, which is then undetectable already on day 3, in parallel with the onset of expression of* SOX17* and* CXCR4*, indicating the termination of the mesendoderm stage and the initiation of the DE stage [19, 27].* SOX17* and* CXCR4* also clustered together at the DE stage, which is expected since* CXCR4* is regulated by* SOX17* [27].* CER1* is typically used as DE marker in combination with other genes, since it is also expressed in mesoderm [28]. Our results indicate that, unlike* SOX17* and* CXCR4*, the onset of* CER1* occurs in the primitive streak, which is in agreement with* in vivo* liver development [29].* HHEX* promotes the further hepatic differentiation by terminating the DE stages, thereby initiating hepatoblast differentiation. The onset of* HHEX* expression is suggested to occur at the DE stage and continue through the hepatoblast stage [30]. Our results demonstrate the upregulation of* HHEX* in the mesendoderm and its expression is terminated at the hepatoblast stage.* HHEX* also cluster with* CER1*, and both of these genes are known to be induced by* SOX17*. This was, however, not reflected in our results, since* CER1* and* HHEX* were expressed before* SOX17*. In addition,* HHEX* is required for the normal expression of* CER1*, which may explain the clustering of this pair of genes with* SOX17* and* CXCR4* [28]. However, the expression of* HHEX* was terminated at the later hepatoblast stage, whereas* in vivo* expression of* HHEX* is maintained throughout the hepatic development [14].

Small fluctuations in the RNA levels were observed as an artifact at each medium change after the DE stage and these were not included in the gene expression and clustering analysis since they would mask the real effects when the gene expression is stabilized and introduce nonrelevant noise. The genes* TBX3* and* HNF4A* are known to be expressed in the hepatic endoderm stage [1, 4, 14, 31, 32]. Interestingly, these genes are induced directly after the application of HEP progenitor medium (2) indicating the efficiency of the medium in promoting the development into hepatoblasts.* TBX3* is proposed to promote hepatocyte differentiation from hepatoblasts and represses cholangiocytes by downregulating* HNF6* and* KRT18* and additionally promotes the expression of* HNF4A*, which is a key regulator of morphological and functional differentiation of hepatocytes [2], by repressing the transcriptional repressor of* HNF4A* [32].* HNF6* is expressed at the hepatoblast stage and promotes the differentiation of hepatoblasts towards cholangiocytes [14, 32]. However,* HNF6* is required for proper liver development in the early stages [33]. Towards the end of the hepatoblast stage,* AFP* expression is detected, which is a fetal hepatocyte marker expressed until birth. Subsequently, it is downregulated but can still be detected in adult liver at very low levels [34]. Our results show that* AFP* is coexpressed in some hepatocyte-like cells with the mature hepatocyte markers* AAT* and* ALB* and is downregulated in the maturing hepatocyte-like cells, however, not to a level below the detection limit of the assay (Figure 6).* PROX1* and* KRT18* are upregulated at day 15 (Figure 7), directly after the switch to HEP maturation base medium (3Ap) and supplement (3Bp).* PROX1* together with* HNF6* was demonstrated to be crucial for complete hepatocyte programming including recapitulating the metabolic functionality* in vitro* [31].* KRT18* is a hepatic marker which we observed to be weakly expressed in hepatoblasts and which was upregulated in later stages (Figure 7), which is in accordance with other studies [35].* AAT* is detected at day 19 at low levels compared to freshly isolated primary hepatocytes.* ALB*, which is a mature hepatocyte marker, is detected at day 20. This gene could also be expressed in nonfunctional hepatocytes when the programming process fails to mimic liver organogenesis [31]. However, the detection of* CYP3A4* mRNA expression (Figure 7) and CYP3A4 immunopositive hepatocyte-like cells (Figure 12) as well as CYP activity assay results that show comparable results to cryoplateable human primary hepatocytes (Figure 18) confirmed the hepatic functionality of the generated hepatocyte-like cells. In addition, the RNA and protein expression of the drug transporters MRP2, OATP1B1, BSEP, and NTCP (Figures 16 and 17), as well as the ability to store glycogen (Figure 19), also demonstrate the hepatic functionality of these cells. The interindividual variation in CYP activity and drug transporters expression in hPSC-derived hepatocytes show similarity to what is typically observed in their* in vivo* counterparts [11, 13]. However, unlike during the* in vivo* liver development [14],* ALB* is not detected in hepatoblasts in our study.

The clustering analysis of the later differentiation stages reveals the shift of the cluster* PROX1, TBX3, KRT18, HNF4A*, and* AFP* at days 7 to 21 to the cluster* HNF4A, AFP*, and* TBX3*, and the cluster* PROX1, AAT, HNF6, ALB, CYP3A4*, and* KRT18* at days 21 to 35. The clustering of* PROX1* and* KRT18* both between days 7 and 21 and between days 21 and 35 could be explained by the upregulation of both genes when switching to the HEP maturation base medium (3Ap) and supplement (3Bp). Importantly,* PROX1* and* HNF6* were described to be involved in the same gene regulatory network controlling the migration and adhesion of hepatocytes* in vivo* [33, 36]. Since* HNF6* and* KRT18* are both regulated by* TBX3* [32] it is plausible that* PROX1* and* KRT18* are involved in the same gene regulatory network. HNF4a is known to bind to more than 40% of liver active genes [2], and its expression is already upregulated at day 8, which could explain its clustering with* AFP* and* TBX3*, which are also upregulated early (Figure 13). At days 21 to 35 there is a clear distribution of mature hepatocyte markers (*AAT, ALB, KRT18*, and* CYP3A4*) into adjacent clusters separated from earlier markers (*AFP, TBX3*). Finally, the clustering of* HNF6* with* KRT18* could possibly be explained by the common regulation by TBX3 [32]; however, the clustering of* ALB* with* HNF6* needs to be further investigated.

## 5. Conclusion

The process of differentiating hPSCs into hepatocytes used in the present study demonstrated highly synchronized gene expression profiles of several lineage-specific genes across 6 hPSC lines. Comparing these data to the results from previous studies of both* in vitro* and* in vivo* differentiated hepatocytes revealed important similarities but also some differences, such as the absence of coexpression of* PROX1* and* HNF6*, which promotes the induction of* HNF1A*. Furthermore, the silencing of* HHEX* at early stages also deviates from the* in vivo* situation. A successful correction of these deviations would have the potential to significantly improve the functionality of* in vitro* derived hepatocytes. Moreover, further investigation of the interaction between* ALB* and* HNF6* could also lead to improvements of future differentiation protocols. Taken together, this study adds yet another piece of information to the efforts of improving* in vitro* hepatic differentiation protocols, thereby bringing hPSC-derived hepatocyte based models to a wider practical use.

## Figures and Tables

**Table 1:** Summary of TaqMan Gene Expression Assays used in the study and on which days of the differentiation process they were applied.

Gene symbol	Product number	Days
ABCB11 (BSEP)	Hs00184824_m1	31, 34
ABCC2 (MRP2)	Hs00960494_m1	31, 34
AFP	Hs00173490_m1	10–35
ALB	Hs00910225_m1	19–35
T (Brachyury)	Hs00610080_m1	0–3
CER1	Hs00193796_m1	1–9
CREBBP	Hs00231733_m1	0–35
CXCR4	Hs00237052_m1	2–10
CYP3A4	Hs00604506_m1	21–35
HHEX	Hs00242160_m1	1–11
HNF4A	Hs00230853_m1	9–35
ONECUT1 (HNF6)	Hs00413554_m1	10–35
KRT18	Hs02827483_g1	13–35
KRT19	Hs00761767_s1	6–35
NANOG	HS2387400_g1	0–7
OCT4	Hs04260367_gH	0–7
PROX1	Hs00896294_m1	14–35
SERPINA1 (AAT)	Hs00165475_m1	19–35
SLCD1B1 (OATP1B1)	Hs00272374_m1	31, 34
SLC10A1 (NTCP)	Hs00161820_m1	31, 34
SOX17	Hs00751752_s1	2–10
TBX3	Hs00195621_m1	7–35

**Table 2:** Summary of primary and secondary antibodies used in the study.

Primary antibody	Class	Dilution	Distributor, catalogue number	Secondary antibody	Dilution	Distributor, catalogue number	Applied to cells day
Albumin (ALB)	Rabbit-IgG	1 : 500	DakoCytomation, A0001	Donkey anti-rabbit-IgG-Alexa 488	1 : 1000	ThermoFisher Scientific, A-21206	24, 25, 29, 30
CYP3A (4)	Rabbit-IgG	1 : 200	Cypex, PAP011	Donkey anti-rabbit-IgG-Alexa 488	1 : 1000	ThermoFisher Scientific, A-21206	29, 31
CK 18	Mouse-IgG1	1 : 100	DakoCytomation, M7010	Goat anti-mouse-IgG-Alexa 488	1 : 500	ThermoFisher Scientific, A-11029	24, 25, 29, 30
HNF4a	Rabbit-IgG	1 : 400	Santa Cruz, sc-8987	Donkey anti-rabbit-IgG-Alexa 488	1 : 1000	ThermoFisher Scientific, A-21206	14, 24, 25, 29, 30
Oct4	Mouse-IgG2b	1 : 200	Santa Cruz, sc-5279	Donkey anti-mouse-IgG-Alexa-488	1 : 1000	ThermoFisher Scientific, A-21202	0, 5, 7
Sox17	Goat-IgG	1 : 500	R&D Systems, AF1924	Donkey anti-goat-IgG Alexa 594	1 : 1000	ThermoFisher Scientific, A-11058	5, 7
SSEA-1	Mouse-IgM	1 : 200	Santa Cruz, sc-21702	Donkey anti-mouse-IgG-Alexa-488	1 : 1000	ThermoFisher Scientific, A-21202	0
AAT	Rabbit-IgG	1 : 200	DakoCytomation, A0012	Donkey anti-rabbit-IgG-Alexa 488	1 : 1000	ThermoFisher Scientific, A-21206	24, 25, 29, 30
*α*-fetoprotein (AFP)	Mouse-IgG1	1 : 500	Santa Cruz, sc-51506	Goat anti-mouse-IgG-Alexa 594	1 : 1000	ThermoFisher Scientific, A-11032	14, 24, 25, 29, 30
BSEP	Goat-IgG	1 : 100	Santa Cruz, sc-17292	Donkey anti-goat-IgG-Alexa-488	1 : 1000	ThermoFisher Scientific, A-11055	29
OATP-C (OATP1B1)	Rabbit-IgG	1 : 200	Santa Cruz, sc-33609	Donkey anti-rabbit-IgG-Alexa 488	1 : 1000	ThermoFisher Scientific, A-21206	29
NTCP	Goat-IgG	1 : 400	Santa Cruz, sc-107030	Donkey anti-goat-IgG-Alexa-594	1 : 1000	ThermoFisher Scientific, A-11058	29
MRP2	Rabbit-IgG	1 : 50	Santa Cruz, sc-20766	Donkey anti-rabbit-IgG-Alexa 488	1 : 1000	ThermoFisher Scientific, A-21206	29
